# Molecular pathological study of the human nasopharyngeal carcinoma CNE3 cell line

**DOI:** 10.3892/ol.2013.1513

**Published:** 2013-08-05

**Authors:** FEI LIU, WEI JIAO, XIANG-LAN MO, JIAO LAN, RUI-PING XIAO, XIANG-ZHEN ZHOU, ZHEN-LU HUANG, XIAO-MIN MO, GANG LI

**Affiliations:** 1 Research Center of Medical Sciences, The People’s Hospital of Guangxi Zhuang Autonomous Region, Nanning 530021, P.R. China; 2 Department of Pathology, The People’s Hospital of Guangxi Zhuang Autonomous Region, Nanning 530021, P.R. China; 3 Laboratory of Electron Microscopy, Guangxi Medical University, Nanning 530021, P.R. China

**Keywords:** nasopharyngeal carcinoma, CNE3, molecular pathology

## Abstract

The present study aimed to identify the molecular pathological changes of the nasopharyngeal carcinoma (NPC) epithelial CNE3 cell line, which has been used in experimental studies for 20 years in a culture environment. The pathological type of NPC and the presence of the Epstein-Barr virus (EBV) were identified. CNE3 short tandem repeats (STRs) were amplified, analyzed and compared using metastatic carcinoma tissue from primary NPC. Immunohistochemistry (IHC) and *in situ* hybridization (ISH) were used to identify the immunophenotype and EBV-encoded small RNA (EBER) expression in nude mice transplanted CNE3 tumor cells. Polymerase chain reaction (PCR) and DNA sequencing were used to identify the EBV oncogene, *Bam*H1-A right frame 1 (BARF1) and electron microscopy was used to analyze the organization of the ultrastructure. CNE3 was not cross-contaminated by other human cell lines and the EBV was no longer present in the CNE3 cells. The pathological type of CNE3 was transformed from an undifferentiated non-keratinizing carcinoma with focal adenocarcinoma differentiation into a poorly-differentiated adenocarcinoma. In conclusion, this knowledge on the molecular pathological changes of CNE3 may aid in the development of new research approaches for NPC.

## Introduction

A study of nasopharyngeal carcinoma (NPC) recently demonstrated that the mortality rate of this disease was increasing in Guangxi, China (1). The current prevention and therapy for the disease does not indicate an optimistic outcome. Since CNE3 was established from a liver metastatic carcinoma tissue of primary NPC (2), it has been used in basic studies of NPC (3–8). Certain studies revealed that the molecular biological characteristics were different between primary NPC and metastatic NPC, including expression of EBV-encoded small RNA 1 (EBER1) (9), zinc levels (10), karyotype and differentiation (11). Therefore, CNE3 may be useful for studies of metastatic NPC. However, the molecular pathology of CNE3 is altered due to long-term culture *in vitro*. The knowledge obtained from the continuing progress in molecular biological technology combined with the present study of the molecular pathology of CNE3 may provide useful data for subsequent studies.

## Materials and methods

### Cell culture

The human NPC epithelial cell lines, CNE1, CNE2, CNE3 and C666-1, were preserved in the Research Center of Medical Sciences, The People’s Hospital of Guangxi Zhuang Autonomous Region (Nanning, China). As a control, CNE3 was obtained from the National Institute for Viral Disease Control and Prevention, Chinese Center of Disease Control and Prevention (Beijing, China).

### Animal experiments

In order to establish the nude mouse tumor model of CNE3 through subcutaneous transplantation, Balb/c pure line mice were obtained from the Guangxi Medical University Laboratory Animal Centre (certification no. SCXK Gui 2009–0002). This study was approved by the ethics committee of The People’s Hospital of Guangxi Zhuang Autonomous Region.

### CNE3 short tandem repeat (STR) loci analyses

The CNE3 STR loci were authenticated using an ABI 3100 Genetic Analyzer (Microread Gene Technology, Beijing, China).

### Histomorphology experiments

The tissues were obtained from a patient’s primary nasopharynx foci in 1982, the same patient’s metastatic liver carcinoma of primary NPC in 1988 and nude mice transplanted tumor in 2012. The tissues were fixed using 10% formalin and paraffin embedding, then sliced and stained with hematoxylin and eosin (HE). An optical analysis was then performed. Subsequent to being double stained with uranyl acetate-lead citrate, the transplanted tumor was observed using a H-7650 transmission electron microscope (TEM; Hitachi, Tokyo, Japan).

### Immunohistochemistry (IHC)

A non-biotin horseradish peroxidase (HRP) ready-to-use two-step detection system (ZSGB-BIO, Beijing, China) and BX51 fluorescence microscopy (Olympus, Tokyo, Japan) were used in the IHC analysis. The positive brown granules, which were more abundant than the unspecific staining background, were mainly distributed in the cell nucleus (p63) or cytoplasm [cytokeratin (CK)5/6, CK7]. The positive cell rates and staining intensities were comprehensively analyzed in the intact slices using high power fields (x200 or ×400). The results of the positive cell rates (<10%) and weak coloring were negative. The results of the positive cell rates (>10%) and dark brown granules were positive.

### EBER in situ hybridization (EBER-ISH)

An EBER-ISH kit (ZSGB-BIO) identified that the positive brown granules were mainly distributed in cell nuclei.

### DNA extraction and polymerase chain reaction (PCR)

DNA was extracted using the Genomic DNA Purification kit (Promega, Madison, WI, USA). The following primer sequences were used for the amplification of *Bam*H1-A right frame 1 (BARF1; NC_007605.1): BARF1 forward, 5′-CCAGGCTGTCACCGCTTTC-3′ and reverse, 5′-CGCCAT TTGCCGCAGTT-3′. The sequence length was 469 bp. The reaction conditions consisted of 12 μl 2X*Taq* PCR Mix (Tiangen, Beijing, China), 0.5 μl template, 0.5 μl forward primer, 0.5 μl reverse primer and 11.5 μl ddH_2_O. The reaction program consisted of an initial denaturation step at 95°C for 10 min, denaturation at 94°C for 35 sec, annealing at 57°C for 35 sec, extension at 72°C for 35 sec for 40 cycles and a final extension at 72°C for 10 min. The sequence was amplified using S1000 Thermal Cycler PCR (Bio-Rad, Hercules, CA, USA).

### Sequencing

Purified PCR products were analyzed by the 3730 automatic DNA sequencer (ABI, USA).

## Results

### Contamination status of CNE3

**Figure 1:**
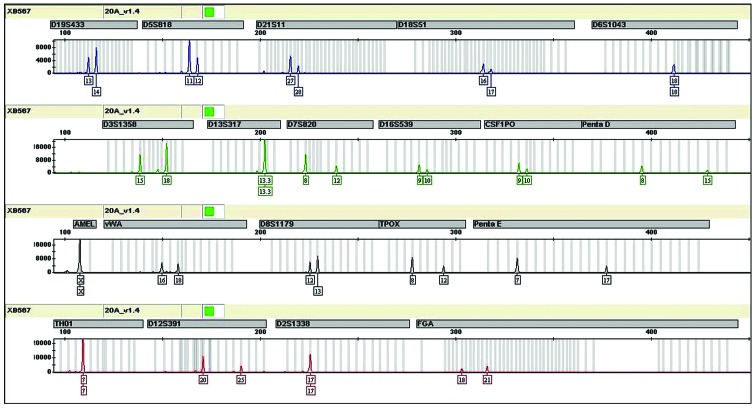
STR loci genotyping of CNE3. The 20 STR loci are not triallelic so the cell line is not cross-contaminated by other human cells. STR, short tandem repeat.

A total of 20 STR loci were not triallelic and the results revealed that CNE3 was not cross-contaminated by other human cells (Fig. 1).

### Nude mouse transplanted tumor model

**Figure 2:**
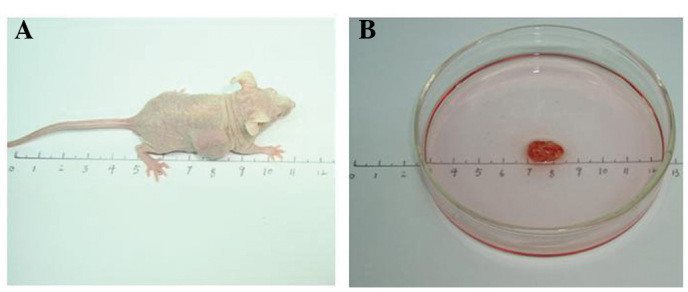
Balb/c nude mouse tumor model of CNE3. (A) A mouse with CNE3 tumor was measured by straight scale. (B) The tumor mass was measured by straight scale after the mouse was dissected.

The transplanted CNE3 tumor volume was 0.15 cm^3^ after 14 days (Fig. 2).

### Adenocarcinoma morphological characteristics

**Figure 3:**
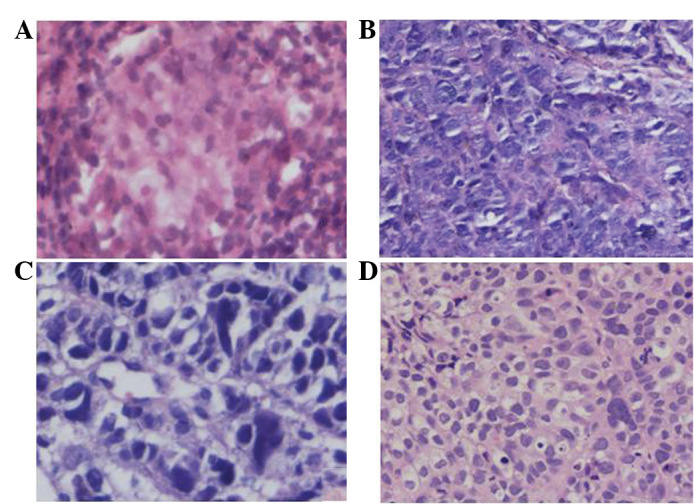
HE staining of NPC. (A) The carcinoma tissues of the primary nasopharynx foci were obtained from a patient in 1982 (x400). (B) Undifferentiated non-keratinizing carcinoma tissues of liver metastatic carcinoma of primary NPC were obtained from the same patient in 1988 (x200). (C) Primitive adenoid differentiation tissues of metastatic liver carcinoma of primary NPC were obtained from the same patient in 1988 (x400). (D) Nude mouse transplanted tumor tissues of CNE3 were obtained in 2012 (x200). HE, hematoxylin and eosin; NPC, nasopharygeal carcinoma.

**Figure 4:**
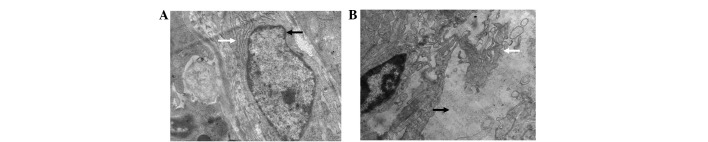
TEM observation of nude mouse transplanted CNE3 tumor tissues. (A) Abnormal nuclei (black arrow) and abundant rough endoplasmic reticulum ranged lamellar structure (white arrow; magnification, ×20,000). (B) Micrograndular cavities (black arrow) and microvilli on the surface of cavities (white arrow; magnification, ×40,000). TEM, transmission electron microscopy.

Microscopically, the cancer cells from the primary nasopharynx foci indicated the structure of an undifferentiated non-keratinizing carcinoma. The cells were polygonal, weakly basophilic, contained a large nucleus with prominent nucleoli, had little cytoplasm and an unclear cell boundary (Fig. 3A), which were arranged in sheets and nests. The cells of the primary metastatic liver carcinoma revealed a primitive adenoid structure. The cells had a circular form, rich cytoplasm and clear cell boundaries (Fig. 3B and C). The cells of the nude mice with the transplanted tumors indicated an adenoid structure. The cells were a spindle or polylateral shape and there were physaliphorous cells (Fig. 3D). Electron microscopy observations revealed the typical characteristics of an adenocarcinoma (Fig. 4).

### IHC results

**Figure 5:**
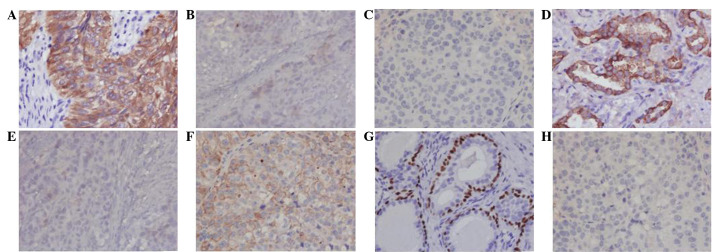
IHC expression of CK5/6, CK7 and p63 in various NPC tissues. (A) CK5/6 expression of squamous epithelial tissues were used as a positive control (x200). (B) CK5/6 was positively expressed in some of the metastatic liver carcinoma tissues of primary NPC (x200). (C) CK5/6 was negative in the nude mouse transplanted CNE3 tumor tissues (x200). (D) CK7 expression from lung adenocarcinoma tissues was used as a positive control (x200). (E) CK7 was positively expressed in some of metastatic liver carcinoma tissues of primary NPC (x200). (F) CK7 was positively expressed in the nude mice transplanted CNE3 tumor tissues (x200). (G) p63 expression from prostatic basal tissues was used as a positive control (x200). (H) p63 expression was absent in the nude mouse transplanted CNE3 tumor tissues (x200). IHC, immunohistochemistry; CK, cytokeratin; NPC, nasopharyngeal carcinoma.

Positive CK5/6 and CK7 results indicated that the metastatic liver carcinoma tissues had features of adenocarcinoma and undifferentiated non-keratinizing carcinoma. The negative results for CK5/6 and p63 expression and the positive result for CK7 expression indicated that CNE3 only had features that were specific to an adenocarcinoma (Fig. 5).

### ISH results

**Figure 6:**
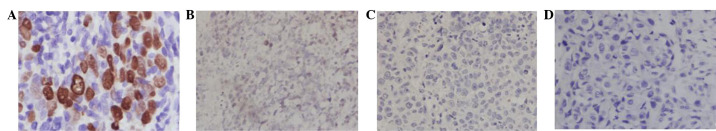
ISH expression of EBER in various NPC cells. (A) EBER of nasopharynx undifferentiated non-keratinizing carcinoma cells was used as a positive control (x200). (B) EBER was positive in some of metastatic liver carcinoma cells of primary NPC (x200). (C) EBER was negative in the nude mouse transplanted CNE3 tumor cells (x200). (D) EBER was negative in the nude mouse transplanted CNE3 tumor cells from Beijing, China (x200). ISH, *in situ* hybridization; EBER, Epstein-Barr virus (EBV)-encoded RNA; NPC, nasopharyngeal carcinoma.

The liver metastatic carcinoma cells were positive for EBER; however, the nude mice transplanted tumor CNE3 cells were negative for EBER. The results indicated that the EBV was no longer present in the CNE3 cells (Fig. 6).

### PCR and DNA sequencing results

**Figure 7:**
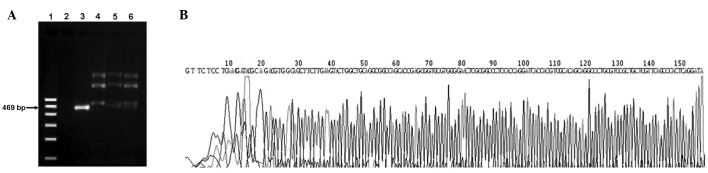
BARF1 PCR and sequencing in various NPC cell lines. (A) Lanes 1–6 represent the DNA marker, negative control, C666-1, CNE1, CNE2 and CNE3, respectively. The DNA markers are 100, 200, 300, 400, 500 and 600 bp. The product length of the BARF1 PCR product was 469 bp. (B) The PCR products of C666-1 were sequenced, but other PCR products were not. BARF1, *Bam*H1-A right frame 1; PCR, polymerase chain reaction; NPC, nasopharyngeal carcinoma.

The results of 3–4 unspecific amplification bands indicated that the EBV was no longer present in the CNE1, CNE2 and CNE3 cells. C666-1 was used as a positive control and ddH_2_O was used as a negative control (Fig. 7A). The PCR products were not sequenced, with the exception of C666-1. The PCR sequence of C666-1 was matched with the BARF1 gene, according to the NCBI blast database (Fig. 7B).

## Discussion

Scanning the tissue slices of the nasopharynx primary foci and liver metastatic carcinoma of primary NPC, the histological type of the nasopharynx primary foci was identified as an undifferentiated non-keratinizing carcinoma. The main area of liver metastatic foci was the undifferentiated non-keratinizing carcinoma structure. However, the other area indicated a primitive adenoid structure. The change suggested that the CNE3 cells were differentiating towards an adenocarcinoma. The CNE3 cell line has had the features of a poorly-differentiated adenosquamous carcinoma since it was established in 1992. EBV has been shown to express specific proteins, including EBV nuclear antigen (EBNA) and latent membrane protein (LMP), in the 19th passage cells of nude mouse transplanted tumors (2). Teng *et al*(4) detected EBV in the 33rd passage cells. CNE3 has been passaged and preserved well for 20 years. The present study used the CNE3 cell line to establish a Balb/c nude mouse transplanted tumor model and then detected the tumor tissues by morphological and molecular pathological experiments. As a result, certain changes were identified subsequent to comparing the nasopharynx primary foci tissues with the metastatic liver carcinoma tissues.

The formerly used single method of pathological detection may cause biases of the morphological diagnosis. Accompanied by a widespread application of IHC, numerous studies have demonstrated that the combined expression of p63 and CK5/6 may improve the diagnostic accuracy of undifferentiated non-keratinizing carcinoma (12). CK7 was highly expressed in the nasopharyngeal glandular epithelium (13). Therefore, a combined application of p63, CK5/6 and CK7 was able to distinguish between undifferentiated non-keratinizing carcinoma and adenocarcinoma. Immunophenotyping analyses of nasopharyngeal undifferentiated non-keratinizing carcinoma were shown to be p63^+^, CK5/6^+^ and CK7^−^. Therefore, the three markers were used to detect the tissues of the nude mouse transplanted tumors. The CK5/6 and CK7 results from the metastatic liver carcinoma tissues of primary NPC revealed that the CNE3 xenograft transformed from an undifferentiated non-keratinizing carcinoma into a poorly-differentiated adenocarcinoma. Electron microscopy further confirmed that the transplanted tumor tissues had classical characteristics of a poorly-differentiated adenocarcinoma, consisting of abundant rough endoplasmic reticulum, a ranged lamellar structure and microvilli on the surface of the micrograndular cavities. The fast growth and predominant quantities of the adenocarcinoma cells may have gradually hampered the growth space of the undifferentiated non-keratinizing carcinoma in the continuing passage.

EBER-ISH is considered to be the gold standard for detecting EBV in cancer cells (14). The metastatic liver carcinoma cells of primary NPC were positive for EBER. However, the nude mice transplanted tumor CNE3 cells were negative for EBER. In 1996, EBV markers of CNE1, CNE2 and CNE3 were detected using ISH, western blotting, southern blotting and PCR. The techniques gave positive results, particularly when using PCR for BARF1. The expression of EBV was strongest in the CNE2 cell line (4). The undifferentiated C666-1 cancer cell line was used as a positive control (15). The conservative carcinogen, BARF1 (16), was identified in order to confirm whether EBV was present in the tissues. The PCR results indicated that the CNE1, CNE2 and CNE3 cells were negative for BARF1, with the exception of C666-1. Therefore, the EBV was no longer present in the CNE3 cells.

EBNAl is a unique viral protein that is found in the four forms of latent infection by EBV. It provides a distinct episome maintenance function by binding to oriP, which is the latent origin of DNA replication (17,18). Therefore, the expression level of EBNA1 is a key factor that episomes maintain in a steady state *in vitro*. If all episomes are lost, continuously mutated or partially missed, EBV will be lost.

The characteristics of CNE3 were studied and the pathological type was confirmed to be a poorly-differentiated adenocarcinoma with a low incidence rate. In conclusion, this knowledge on the molecular pathological changes of CNE3 may aid in the development of new research approaches for NPC.
